# Can Māori Negotiate School Attendance in State-Led Education in Aotearoa, New Zealand?

**DOI:** 10.1007/s40841-023-00283-6

**Published:** 2023-04-06

**Authors:** Sarah-Kay Coulter

**Affiliations:** Faculty of Education and Social Work, The University of Auckland, Auckland, New Zealand

**Keywords:** Education, Indigenous rights, Children, Sustainable development goals, Treaty of Waitangi

## Abstract

There is a conflict between the claims of Māori sovereignty and the imposition of State legislation on Māori children. This conflict of interest has been given very little consideration in the public sphere. This research-informed article speculates that despite legislation ensuring that education attendance is fixed as a legal obligation for all Primary and Secondary aged children, there is urgency to address if conceivably this is a deeply flawed assumption as it contradicts notions of tribal sovereignty. Cautiously, this article does not romanticise past positioning of Māori peoples, nor makes claims to indigenous righteousness, rather moves to suggest that State-led education belongs part of positive outcomes for Māori, however there must be negotiation to the terms and expectations for education attendance. This paper is a catalyst for future orientated discussion aiming to broaden what education can move to become in Aotearoa, New Zealand.

## Introduction

In the Statement of Intent for Crown Law (2021) it states: ‘Te Ao Māori and Te Tiriti sit at the heart of everything we do at Crown Law’ and this discussion highlights the complexities associated Māori sovereignty and the imposition of State legislation on Māori children in the context of mandating school attendance in Aotearoa, New Zealand. It seeks to demonstrate past notions for education as evidenced in traditional Māori societies and traces the colonial mindset toward education as evidenced in law as society developed. This discussion considers education in a postcolonial era as transitional, and the negotiation of terms ensures both the State and Māori peoples can clearly articulate aspirations. Key pieces of domestic and global legislation are considered to highlight the tensions associated with contemporary manifestations of indigenous rights. This paper ensures that education in an indigenous-settler-globalised nation continues to develop and suggests that Māori peoples along with the State can find effective ways to negotiate the terms of attending school in Aotearoa, New Zealand.

## Māori Perspectives of Education

Education plays an important role in society as it equips both cultures and communities with the knowledge and skills required for human survival and social flourishing. Each culture of the world has developed patterns of human relations, embedded with education ideologies, knowledge dissemination, transfer of skills and capabilities to ensure the survival of the collective across time. The first inhabitants of the landmass of Aotearoa, New Zealand are the collective known as Māori or tangata whenua. Prior to European settlement, the social order of tangata whenua was intergenerationally defined and includes distinct relational characteristics as it centered human and non-human interconnectedness. Human collectives could be identified within iwi (tribe), hapū (sub-tribe) and whānau (family) groups. This social system ensured survival and developed in line with ethics and moral intent of the collective. Importantly, tribal constitutionalism was not static, nor fixed and permanent, as dynamism, authority and adaptation were a significant feature of the approach to social order. Judge Caren Fox (2010) highlighted that Māori were familiar with the notion of political autonomy and describes that such a structure was long developed, fluid and malleable that provided a natural rhythm and order to Māori society.

Whānau, was a site of authority within the tribe, a small, nimble entity, extending beyond the nuclear family to include kaumatua and kuia (tribal elders), with sisters and brothers, aunts, uncles and all children. Esteemed tribal elder, Jackson explained at a *Unity Books* event in Wellington that this entity was mokopuna (grandchild) centric. He shared that a unique value of tribal constitutional arrangements was the ‘individual child’ was the central force of both social and tribal life and by birthright, a mokopuna belonged part of the political aims, social structures and dynamics of the whānau (Unity Books, 2022). Walker (1990) suggests that purpose of the whānau was:

…the procreation and nurture of children. In the absence of parents engaged in gardening or other activities related to the food quest, all other adults in the vicinity were in loco parentis. This meant that in the whānau children were used to receiving care and affection from many people besides their Parents. (Walker, 1990, p. 63)

Children seen as part of the collective resources, the wealth and livelihood of the group and as such were protected, maintained and nestled within the whānau, arguably the center and the linkage to all functioning of tribal constitutionalism. The unique position of a child reinforced kinship and intergenerational links and the model of childrearing was developed through shared parenting methods reflected in the pūrākau of Maui (a demi-god figure), as he was raised by his grandfather, Tama-nui-te-rā (the Sun). Importantly, the care of mokopuna was firmly entrenched in the social world of the community. It was the family group that were entrusted to know how best to raise, train and ensure all aspects of their being and becoming in the world were part of and contributing to the culture to which they were born into. A child was and remains the most treasured possession in Māori society-so much so, all tribal constitutional arrangements were built around their wellbeing and livelihood. The child as a significant part of functioning for society and from time-to-time collectives made decisions as to ways in which both individual and collective mana (prestige) could be enhanced. This saw the child as a highly esteemed gift with skills and capabilities to be allocated in ways that benefited the welfare of both the individual and to advance the collective.

Considering how education can be conceptualized within tribal constitutional arrangements, it is relevant to suggest that approaches to learning were entangled in localized goals of survival. Systems of learning were developed in accordance with genealogy and bloodlines of which rites of passage or learning experiences were reserved and specialized for specific genders or families within society. For example, one warrior may train on a specific piece of whenua the art of Mau rākau (Māori martial arts), another warrior of a different family may not access too. Similarly, education was tribally nuanced, as learning activities were entangled in land, genealogy, resource rights, some of which could overlap on the same territory. All learning was steeped in the stories of place, migration, mobility, and knowing of all that had come before. Education was therefore embedded within the daily life of culture, localized and intentional, however in some instances children were provided exclusive, highly specialized learning opportunities. An example of this was children who were highly ranked could be raised in whānau groups to learn specific skills or be raised to gain specific knowledge (Graham, 1948). Such practices can be referred to as whāngai, that Mead (1997) translates to mean to ‘to feed or nourish’ ensuring that the child is nurtured to their fullest potential (McRae and Nikora, 2006). Whāngai practices were seen as reciprocal gifts, a political gesture between tribes for cohesion, peace and with future oriented intention for the whānau. The action a promise of unity amongst tribal groups demonstrating the cultural significance of a child, not in an exploitive manner, but rather the contrary, uplift mana through the generosity of exchange. Importantly, in such instances learning experiences outside of the family were not rejected but highly valued.

### Changes to Māori Education

Long established methods and approaches for educating a child were abruptly disrupted with European settlement. It has been widely documented that Aotearoa, New Zealand was colonised and this action has had and continues to be contribute detrimental outcomes for tangata whenua across all spheres of society including education, health, housing, gender and wellbeing (Durie, 2003, 2012; Mikaere, 2017). Colonisation is identified as an imposition of British Imperial establishment, part of a globalized agenda entrenched in political ideologies of conquest, territory, power and control (Smith, 2012). The beginning of this global enforcement attributed to Cook’s Endeavour expedition dated October 1769 (Barnes & McCreanor, 2019). Since this time there have been radical changes to the function and purpose of tribal constitutionalism and a by-product is the positioning of the child in the social order of the shared State of Aotearoa, New Zealand. The child that once solely belonged as the nucleus of family, centralising tribal life, has become entangled in notions of a shared contemporary State, part of and contributing to the goals and intentions of wider Aotearoa, New Zealand.

A unique feature that defines the independence and interdependence of this relationship is the contractual agreement of *Te Tiriti o Waitangi, 1840*. This contract provides an overview of the agreed roles, rights, responsibilities and duties of this arrangement, giving insight into the rights of citizenship and how interior resources would be managed and allocated (The Ministry for Culture & Heritage, 2017). The document declared New Zealand as a ‘Crown colony’ and was a political statement exercising The Queen's governance status in New Zealand, a proclamation of place, position and authority to make provision for settlement, imbued with the conceptual understandings of both divine law and human law, however significantly this document ensured Māori rights to sovereignty. The signatures mark an agreement which endures as a domestic treaty: a foundational commitment and enduring obligation across two groups. Smith (2003) expresses *Te Tiriti o Waitangi, 1840* as the ‘main reference point for the recognition of respective roles, responsibilities and authority over cultural heritage, values and tradition’ (p. 41). It was (and still endures) as a contractual, yet paradoxical agreement that made provision for representatives of the Queen to make rules about behavior and see that everyone obeys them and provide assurance that Chiefs would keep control of their lives and those they represented.

After the signing of the document, legislation was formed that evidenced colonial logics and shortly after there is evidence of the formalized State-led education sector for Aotearoa, New Zealand emerging. The government created legislation for the sector which mirrored the thinking of The Crown and settler population at the time. *The Native Trust Ordinance 1844* highlighted quite explicitly that the intention of education was for the native to be civilized with speed into the ‘colonial’ lifestyle, notions of Māori sovereignty not evidenced in the text.

An Ordinance for appointing a Board of Trustees for the Management of Property to be set apart for the Education and Advancement of the Native Race…. Whereas the Native people of New Zealand are by natural endowment apt for the acquirement of the arts and habits of civilised life, and are capable of great moral and social advancement: And whereas large numbers of the said people are already desirous of being instructed in the English language and in English arts and usages: And whereas great disasters have fallen upon uncivilised nations on being brought into contact with Colonists from the nations of Europe, and in undertaking the colonisation of New Zealand Her Majesty's Government have recognised the duty of endeavouring by all practicable means to avert the like disasters from the Native people of these Islands, which object may best be attained by assimilating as speedily as possible the habits and usages of the Native to those of the European population.

*The Education Act 1847* was passed as law only three years later and proclaimed order and intention for the education sector. However, there is no mention of ‘natives’ nor ‘aboriginals’ recorded within this statute and without context it is hard to gauge if this was intended or unimportant. Dorsett’s (2017) shares one explanation suggesting that ‘the British…. did not intend Māori to be subject to British law in all circumstances’ (p. 3) regardless, Lee and Lee (1995) report Māori at this time were increasingly dissatisfied with assimilation practices, as Māori peoples were ‘subjects’ of the law, ‘the Native people’ continually placed in a social structure that didn’t affirm chiefly authority, instead a subordinate class. The social challenges experienced by Māori peoples in the newly established social structure continued to be felt acutely as during this time the population expanded with increased numbers of settlers. The position of the child, simply seen as property of the State, entangled in intent of the wider goals of the wider population. This was a time of social mobility for the settler population, but for tangata whenua, tribal retention of tino rangatiratanga (absolute sovereignty) over lands and settlements, and autonomy of taonga katoa (tribal treasures) was fraught with sacrifice and struggle.

It was *The Education Act 1847* that made the provision for public funds to be used for education (one twentieth part of the estimated revenue) as well as school establishment and outlined the roles and responsibilities of inspectors, management and teachers (Lee & Lee, 1995). With tensions emerging that tangata whenua were dissatisfied with the ways in which Aotearoa, New Zealand was progressing, two decades after the *Education Act 1847, The Native Schools Act 1867* made provision for the establishment of a system of secular village primary schools, this under the control of the Department of Native Affairs and instruction for ‘natives’ was to be conducted entirely in English. This legislation re-established the government's intent to assimilate Māori into the dominant society (Simon, 1998; Simon & Smith, 2001). The inspections that were carried out of schools during this time also reported on the work of civilizing the native race (Taylor, 1983). It was at this point, education was free but not compulsory (Calman, 2012; Simon, 1998). When the *Education Act 1877* mandated student enrolment in education, initially this only applied to the settler population, not Māori. But by the fiftieth year of colonial involvement in education in Aotearoa, New Zealand, *The School Attendance Act 1894* passed as law, ordering education was compulsory for all youth of New Zealand, inclusive of tangata whenua.

## Mandating School Attendance

*The School Attendance Act 1894* gives only four scenarios as to why a child may not attend a school, such as sickness, lack of access and non-attendance had to be ‘agreed to’ by colonial officials. The Act demanded all those between the ages of seven and thirteen to be required to be at ‘school’ and the failure to do so was framed by a discourse of ‘neglect’ with the threat of judicial summons. Arguably, this action poses a sizable conflict with *Te Tiriti o Waitangi, 1840*, given an iwi (tribe), hapū (sub-tribe) and whānau (family) had the right to allocate how possessions and treasures were to participate in the shared economies of a developing social State of Aotearoa, New Zealand. When the *School Attendance Act 1894* was legislated, it suggested the Crown can and does make ‘claim’ to the property of mokopuna Māori. More specifically, the very action of mandating attendance can be seen to contradict the ‘chiefly authority’ and tino rangatiratanga of Article 2 of *Te Tiriti o Waitangi, 1840*. Could the State impose how mokopuna Māori, as a human resource and possession of a tribe, be directed by colonial force? Could or should the government reasonably enforce such a law?

Article 3 of *Te Tiriti o Waitangi, 1840* allows for mokopuna Māori to hold a right to dual forms of citizenship, but mandating education is a ‘right’ and privilege associated with the developing settler-indigenous State; but this is not seen as a duty. Importantly, a duty differs from a legal obligation, as a duty cannot always be enforced by the law, more so a duty is something we ‘ought’ to do. It is challenging to establish through which lens the Crown saw education, but subsequent punitive measures demonstrate education in Aotearoa, New Zealand is a legal obligation. The question remains, how could such an order be reasonably be applied to mokopuna Māori as the Crown did not have ownership or possession of this resource as education is what ‘should’ be done as a contributing citizen toward goals of nationhood, but to order non-attendance as a crime, such a notion becomes problematic. To ‘order’ the way mokopuna Māori could participate in education, potentially prohibits chiefly authority and the jurisdiction of whānau as this this was a possession situated within tribal constitutional arrangements and therefore, all children protected by *Te Tiriti o Waitangi, 1840*. The act of legislating school attendance suggests the Crown has the right to claim the tribal child, mandating how and where this ‘subject’ would be at a particular time. This inherently demonstrates the thinking of the time, that The Crown felt a superiority over Māori subjects, colonial law a way to direct and control property.

Despite any conflict between the roles, responsibilities and authority between tribal understandings and the State and its intent for education, between 1877 and 1964 education continued to change in line with social expectations for publicly funded compulsory education (primary and secondary education). A report by McGuinness Institute Limited (2016) who work towards a sustainable future for New Zealand mentions that one of the intentions of State-led education was that all citizens would be able to have access to equal opportunities.

While it could be argued that the goal of equality of opportunity has been present since the Education Act 1877 was implemented, that opportunity was much more narrowly focused than it is today. Equality of opportunity as an aim in education was publicly articulated in 1939 by then Prime Minister Peter Fraser, and this has remained an underlying goal in subsequent statements of educational policy (p. 3).

*The 1938 Declaration on Education,* echoes the ideological assumption as education was not just a privilege, but all citizens had an entitlement to education. McDonald (2002) quotes an early speech from Dr C.E. Beeby The Minister of Education from the 1940s:

The government’s objective broadly expressed, is that every person, whatever his level of academic ability, whether he be rich or poor, whether he live in town or country, has a right, as a citizen, to a free education of the kind for which he is best fitted, and to the fullest extent of his powers.

Post World War efforts ensured that as Aotearoa, New Zealand developed the ideological assumption of equal educational opportunities which went largely unchallenged as this was beneficial for the nation State. Over time it became clear that the State-funded education system was formed to replicate colonial practices and comprehensive schooling. Mandating schooling continued for Primary and Secondary students, but to continue this for tangata whenua appears somewhat presumptuous given there is an assumption that Māori subjects would solely want what the State provided, despite a fundamental conflict with the values and approaches to learning pre-colonisation. Although the State was potentially well intended in its desire to create an education system that aimed to socially benefit subjects and create peace and harmony, build skills to contribute to enterprise, publicly funded education was largely oriented by the economic goals of the government. What is most problematic, is that through this window of time, it was Māori peoples that continued to be ‘subjects’ of the law, unseen in the design of the system, nor having any mechanism to discuss, or negotiate the terms and conditions of involvement. This demonstrated by the colonial structure of law making, the methods of teaching, approaches to learning, ideologies underpinning the sector, and education statutes that created a publicly funded system that dictated the one and only way to educate children. Importantly, it was up the 1980s colonial ideas were entrenched and the system changed very little, and it was assumed that education would continue to be provided for, children mandated to attend and would remain funded and controlled by the State (Codd & Openshaw, 2005).

It was only after *The Treaty of Waitangi Act 1975* was passed as law, that all newly developed legislation was to include a reference to New Zealand’s foundational intentions. However, the extent of sector enactment of the ideals embodied in the clause appear murky, given this action emerged from a backdrop of assimilation, race-based policies, bias, assumption and conflict (Hetaraka, 2022).

### Flax Roots Fight by Māori for an Alternative Approach to Education

It was during the 1970s there were movements beginning ‘outside’ the formal structure of publicly funded education which demonstrate a flax-roots push for a change in the systems of education and learning. With support of the Māori Affairs department, Te Kōhanga Reo (Māori-language preschools) evolved at a local level to immerse children in traditional education practices that centred on community development. From 1985 Māori-medium schools known as Kura Kaupapa emerged as a suitable pathway of learning for children who had been immersed in Te Kōhanga Reo and this signaled the State could not exercise full plenary power of mokopuna Māori. The original founders of the movement were strategic in their collaboration to gain public funds and through negotiation and clever thinking the founders demonstrated how The Ministry of Education [MoE] became involved to support the establishment of providers. This then saw Māori Medium Education falling within the legislative intent of *The Education Act 1989*. The MoE entrusted with terms of reference to support the development of mokopuna Māori that required the ‘active protection’ of the learning pathways, however since then it has been found that these obligations did not have follow-through as documented in *Wai 2336: Matua Rautia: Report on the Kōhanga Reo Claim* (The Waitangi Tribunal, 2013). Problematically, education legislation continued to mandate schooling in Aotearoa New Zealand, but there was a missed opportunity by the State to demonstrate a sharing with tribal authority, as the control of rules and requirements for publicly funded education remained firmly with the government. A significant aspect of Māori medium immersion schooling is that since its beginnings there has been a cultural shift in mainstream expectations of the Māori language and Māori culture. Arguably, this movement is a key contributor to revitalization of te reo Māori and present day there is a resurgence of both tribal and national social identity. Alongside this, there has been the rise of domestic employment opportunities for Māori and on a broader scale, indigenous models toward land care and approaches to health are becoming better valued.

### Mandating Attendance: the Legacy Continues

Considering that all schools and education providers are governed by the laws of *The Education and Training Act 2020* [The Act] currently the statute adopts a signaling approach that attendance remains compulsory for students between 6 and 16 in Aotearoa, New Zealand. School Boards (who are seen as the governing entity of each school) must take reasonable steps to ensure its enactment and principals, teachers, parents and guardians are also entangled to which they are subject to the law that mandates regular school attendance. It is important to highlight that time in which one is in compulsory education has increased significantly since the *School Attendance Act 1984* as an additional four years and a weekly increase of an additional 10 ‘opening’ hours for students is now seen.

Present day, the Ministry of Education [MoE] is the government agency with responsibility and stewardship for The Act who ensures government goals are enacted. The MoE suggests attendance for Primary and Secondary education will ideally be at 90%; however a recent report showed that In Term 2 of 2022, 39.9% of students attended schools regularly (Education Counts, 2022). This statistic is far from the 90% goal, given the implications for the schooling sector greatly affected by public health measures of COVID-19. This meant many children experienced irregular attendance due to sickness, isolation and health impacts. Despite this, the legislation remains enforceable for students, schools and boards and regular attendance guidelines are in place.

If there is no reasonable explanation of a child being absent in schooling, by law all subjects are subject to Section 49 of The Act which gives power to boards to appoint constables and/or attendance officers to detect and detain students who are not at school and if deemed appropriate. Students are then subject to punitive measures such as detentions, principal's office visits, suspensions and risk being ‘charged or prosecuted,’ the penalty for non-attendance set at $30 for every school day on which the offence occurs, not exceeding $300 for the first offence, $3000 for second or subsequent. There has been a steady decline in the enforcement of this within the judicial arm of government, with only one recent case *R v Snodgrass [2017]* that court resulted in a conviction, but not a monetary fine, rather the judge reminding the parent of the importance of education. Data from The MoE has captured the ongoing decline of regular attendance in New Zealand schools year-on-year (Education Counts, 2022). Crucially, Māori subjects seem to be particularly high in non-attendance, but arguably as an imposed idea, can we reframe that mokopuna Māori can negotiate attendance?

## External Scrutiny: Indigenous Rights and Human Rights

The trend of non-attendance has been amplified post-pandemic and this prompted the Ministry of Education [MoE] to respond with both an *Attendance and Engagement Strategy* and significant monetary investment administered to school communities to explore ways to get children back to school (Ministry of Education (MoE), 2022). A nationwide Campaign was launched to support school attendance: *Every School Day is a Big Day* which has invested 80 million dollars into initiatives that support children to attend (The New Zealand Government, 2022a). However, as the world is increasingly globalized, what is provided when they attend has been scrutinized as a recent report suggests that the New Zealand education sector is one of the worst in the developed world for child wellbeing (UNICEF, 2020). With reported high rates of obesity, alarmingly high suicide rates in the youth population of Aotearoa, New Zealand and declining proficiency in literacy and mathematics is the structure of education appears failing in its core requirements associated with equity and human flourishing? A cabinet paper prepared by the Social Wellbeing Committee (2021) delivered to the Office of the Minister of Education reinforcing concern, suggests:

New Zealand’s education system performs well for many children and learners, but there are also many who are not served well by our current system, particularly Māori and Pacific learners, those with disabilities and/or learning support needs, and those from disadvantaged backgrounds. (p. 1)

Although such reports potentially overlook the great gains made in many areas of social and cultural growth, the point is the increased scrutiny by both domestic and international stakeholders. As the government has obligations to the Commonwealth and a relationship to the United Nations [UN] Aotearoa, New Zealand is entangled in the aspirational goals of global governance. Education is affected, as with multiple domestic and global stakeholders, who then is responsible to advocate for mokopuna Māori and who retains authority in the discussion? This is a complex and contested field, which Albow (2012) suggests Law remains a political and religious intervention, a proposition of normative order that must still allow for individuals the right to appeal. Aotearoa, New Zealand is a relatively small nation in comparison to other nations of the commonwealth and education goals reiterate the importance of global citizenship, multilateralism, partnerships, geopolitical relations, as in order to fully participate in the world there are attempts to educate children toward goals of ‘promoting peace and security, trade, and human rights, ending hunger, preventing health epidemics, tackling climate change, and protecting the environment’ (New Zealand Foreign Affairs & Trade, 2022). The education system has been developed to enable domestic learning opportunities, but is also duty bound to the world and its complexities, yet how do minority groups such as those who whakapapa Māori as indigenous peoples of world, negotiate such terms of participation? Can involvement remain as something ordered, invited, a moral duty, a privilege, an entitlement or negotiated? What are the mechanisms for discussion?

### Human Rights Law

There has been an increase in human rights laws both domestically and internationally to allow for groups to discuss and debate their involvement and participation in the shared State. *The New Zealand Bill of Rights Act 1990 [BORA]* and *The Human Rights Act 1993* aim to ‘protect and advance’ the rights of ‘minorities’ and *BORA* suggests: “(a) to affirm, protect, and promote human rights and fundamental freedoms in New Zealand; and (b) to affirm New Zealand's commitment to the International Covenant on Civil and Political Rights.” If terms are to be negotiated for State-funded education, such key pieces of legislation may encourage the lens of indigenous rights and human rights to be understood more effectively. Yet, there is always a danger that could lead to the essentialization of whānau, hapū and iwi interests. Although human rights frameworks potentially connect Māori peoples with others that can share a similar experience as indigenous, one concern that emerges is does this become further removed from the highly localized and specific understandings of tribal sovereignty, forever subject to ‘outside’ speculation and enforcement (Erueti, 2017).

*The United Nations Declaration on Indigenous peoples (2007)* declares that those that are indigenous can belong within an international structure and the document has been described as a “landmark” achievement for indigenous peoples (Erueti, 2017) as it claims to be the only instrument to see human rights through an indigenous lens (Anaya, 2009). As a capstone piece of international law of which to grapple with and implement, the extent to which this holds legal effect continues to be debated (Anaya, 2009; Eureti, 2017). Although a cultural relativist would argue that the practices must be viewed from within the culture, not of that of an observer-critiquing the law from this vantage would imply that universal standards are not possible, nor a universal understanding of ‘human rights’ or ‘morality.’ Despite this, the United Nations Committee on Human Rights assessed New Zealand’s compliance with international standards on equality and freedom and *The United Nations Declaration on Indigenous peoples* to share that indigenous groups must be consulted in all decision making-which would be inclusive of statutes, bills and inquiries, yet there is little clarity as to what this means, as conceptualisations of Māori sovereignty are quite different to consultation of agendas with Māori peoples. This distinction is critical as who exercises the plenary power with rights to decide the duties of mokopuna Māori and what agendas are advanced?

Pryor and Bell (2013) prepared a *‘Review of New Zealand’s Constitutional Arrangements’* on behalf of the The Human Rights Commission and each Article of *Te Tiriti o Waitangi,* was defined in the English, in te reo Māori, and then the English translation alongside International Human Rights standards and it was suggested indigenous peoples have an entitlement to the human right of self-determination. However, how is such a standard understood in the context of school attendance, given this appears this aspect of the Law remains imposed, as a non-negotiated norm. *The United Nations Declaration on Indigenous peoples* is part of the comprehensive international and domestic architecture of human rights law, it is presumed that Māori progress and rights to self-determination should have been seen in other aspects of society since these were integrated. Quite the contrary, Anaya (2011) prepared a report titled: *Report of the Special Rapporteur on the rights of indigenous peoples, The situation of Maori people in New Zealand* for the United Nations about progress, however he identified the inadequacies of basic human rights situations for Māori subjects. He summarised;

…the Special Rapporteur cannot help but note the extreme disadvantage in the social and economic conditions of Maori people in comparison to the rest of New Zealand society. (Anaya, 2011, p. 2)

More recently, Mutu (2020) identifies that despite the 2019 Universal Periodic Review conducted by the United Nations [UN] Human Rights Council which made 194 recommendations, of which 48 referenced the human rights of Māori, The Crown advised the United Nations that it accepted 39 of those recommendations, including all those relating to implementing *The United Nations Declaration on Indigenous peoples* (UNGA, ). Mutu (2020) shared that the recommendations have appeared in a number of reports, but they have had little or no improvement. A further global intervention that does give accountability to government aiming to promote indigenous development is reporting on the Sustainable Development Goals [SDG] to which all UN member States adopted the 2030 Agenda, a blueprint for sustainable development that includes 17 goals. The Ministry of Education is responsible for reporting on SDG 4, that focuses on ‘ensuring inclusive and equitable quality education and promoting lifelong learning opportunities for all’ of which the ideologies of equity for education continue to be promoted at a global level, but such goals are not legally binding, but countries are expected to report on their implementation. Aotearoa, New Zealand presented its first Voluntary National Review report to the UN in 2019 demonstrating its progress but acknowledges the SDG 4 has considerable work to do:

We acknowledge that our education system has underserved Māori learners and work is ongoing to support equitable access, inclusion and outcomes. We are committed to continuing efforts to support Māori to participate, achieve and enjoy education success as Māori. (New Zealand Government, 2019, p. 39)

### Freedom of Choice

There have been attempts by government to ensure rights of Māori ‘subjects’ are properly realized in education as children remain entangled as a shared resource of the State, but here lies the possibly: if the terms of participation in State-led education are to be re-negotiated, the removal of attendance mandates, what emerges in this place? What could education of mokopuna Māori become in a postcolonial era? How, who and what are the terms of schooling to ensure that children can remain educated in ways that are intergenerationally defined, inclusive of knowledge that is oriented in tribal constitutional understandings and ensures highly specialized and domestic learning opportunities, while having access to learning to meet the demands to freely participate and contribute to both Aotearoa, New Zealand and global world economies? How can the position of the child which was once the center of all social function remain valued and contribute wholly to future flourishing, locally, nationally, internationally and intergenerationally? Can such an imagined, future oriented possibility become real? Is it time for the State to identify that the legislative burden, the administrative activities of school attendance, the cost, the singular focus of being ordered to participate, be reconsidered to explore the resources and assets of the sector in its current form, to bring forward modern interpretations of Māori sovereignty in education and liberate and open space to possibly?

There are notable examples of Māori sovereignty in action and it is important to highlight that the negotiation of school attendance does not permit a mechanism to turn away completely from the State education system as mokopuna Māori have intergenerational rights to participate in varied learning opportunities. Examples of negotiation in a contemporary context show Iwi groups ensuring a formal relationship agreement is in place with Ministry of Education enabling annual discussion, ensuring perspectives for education are seen, heard and realised (Deed of Settlement, 2021). At a whānau level participation in schooling is negotiated to see mokopuna Māori attending both English Medium and Māori Medium Education pathways concurrently demonstrating a sovereign approach to school attendance (Coulter, 2022). At a hapū level, there are numerous initiatives to connect tribal citizens to peoples and places of the world (Centres of Asia Pacific Excellence, 2022). At a State level, the government of Aotearoa, New Zealand with the Canadian government have formed an Indigenous Collaboration Arrangement to advance and share in the objective of creating and sustaining better outcomes for indigenous peoples (The New Zealand Government, 2022a). But how do such initiatives have potential if mokopuna Māori remain rigidly locked inside attendance expectations in the State-led system? Education plays a critical role in the development of mokopuna Māori, but the heart of this argument is without discussion of the terms of school attendance, has the opportunity been missed to keep bettering the future?

## Conclusion

This discussion highlights that education in Aotearoa, New Zealand has been underpinned by goals of equity, access and including mokopuna Māori in the broad sweep domestic and globalized goals for learning. This paper puts forward a compelling argument that considers the complexities associated with mandated school attendance and the discussion challenges the stability of legislating such a notion. By rigorously exploring *Te Tiriti o Waitangi, 1840, The School Attendance Act 1894, The New Zealand Bill of Rights Act 1990, The Human Rights Act 1993* and The Sustainable Development Goals mokopuna Māori are seen to have both rights and unique opportunity to negotiate the terms of State-led education in a contemporary context. The discussion looked back to conceptualizations of education that was once situated within tribal constitutional arrangements and discovered local and highly specialized pathways for learning and query how Māori sovereignty applies to school attendance in a contemporary context. From the discussion, it seems somewhat dangerous to expect that attendance mandates continue as the discussion highlights that mokopuna Māori remain as subjects controllable by the State, situated within an imposed colonial system, rather than sovereign actors, shaping, making and adapting meaningful learning pathways. It is timely to consider if ordering schooling is deeply flawed to suggest tangata whenua can work with the State strategically to align interests to ensure that the greatest tribal taonga of all, mokopuna Māori, flourish in ways that enrich today, tomorrow and in the generations to come.
